# Navigating COVID-19: Communication and Coordination Between Home Care Agencies and Aides Caring for Older Veterans

**DOI:** 10.1093/geroni/igab046.837

**Published:** 2021-12-17

**Authors:** Emily Franzosa, Kimberly Judon, Eve Gottesman, Tessa Runels, Nicholas Koufacos

**Affiliations:** 1 Icahn School of Medicine at Mount Sinai, Icahn School of Medicine at Mount Sinai, New York, United States; 2 James J. Peters VA Medical Center, Bronx, New York, United States; 3 US Department of Veterans Affairs, Bronx, New York, United States; 4 West Haven VAMC, West Haven, Connecticut, United States

## Abstract

Home health aides are essential members of the home care team, but often report limited communication with agency supervisors. To explore the impact of COVID-19 on these dynamics, we conducted semi-structured interviews with providers (n=9), contracted home health agencies (n=6), and aides caring for veterans (n=8) at an urban Veterans Affairs medical center. Data were analyzed through thematic analysis. Agencies relied on aides to observe and report on patients’ conditions, including COVID-19 symptoms, but aides were not always aware of follow-up and wanted more information about their patients’ health and COVID-19 status. Agencies also reported providing personal protective equipment (PPE) and infection prevention guidance to aides; however, some aides reported purchasing their own PPE and seeking out private COVID-19 testing. Supporting aides by providing needed training and protective resources, and engaging them more collaboratively in medical care, may help improve job satisfaction and quality of care.

